# Contrasting Evolutionary Trajectories: Differential Population Dynamics and Gene Flow Patterns in Sympatric *Halimeda discoidea* and *Halimeda macroloba*

**DOI:** 10.3390/biology14121782

**Published:** 2025-12-13

**Authors:** Yichao Tong, Wei Liu, Yuqing Sun, Jinlin Liu, Qunhui Yang

**Affiliations:** 1 Institute of Marine Science and Technology, Shandong University, Qingdao 266237, China; yctong120@163.com; 2 School of Environmental and Chemical Engineering, Shanghai University, Shanghai 200444, China; hsliuwei@shu.edu.cn; 3 College of Oceanography and Ecological Science, Shanghai Ocean University, Shanghai 201306, China; yuqing_sunn@126.com; 4 State Key Laboratory of Marine Geology, Tongji University, Shanghai 200092, China; 5 Project Management Office of China National Scientific Seafloor Observatory, Tongji University, Shanghai 200092, China; 6 School of Ocean and Earth Science, Tongji University, Shanghai 200092, China; 7 Laoshan Laboratory, Qingdao 266237, China

**Keywords:** *Halimeda*, Xisha (Paracel) Islands, genetic diversity, species diversity, population structure, evolutionary history

## Abstract

Calcareous tropical green macroalgae of the genus *Halimeda* are key reef-builders, yet the drivers of their diversification and population dynamics remain poorly understood. This study analyzed the species diversity of *Halimeda* in the Xisha (Paracel) Islands based on *tuf*A gene sequences, focusing on evaluating the genetic diversity, population structure, and historical dynamics of two widespread species—*Halimeda discoidea* and *Halimeda macroloba*. The results indicate new records of *Halimeda cylindracea* and *Halimeda* cf. *stuposa* in the Xisha (Paracel) Islands. More importantly, *H. discoidea* and *H. macroloba* exhibited significantly different evolutionary histories. Specifically, *H. discoidea* showed a highly fragmented population structure, restricted gene flow, and a multimodal mismatch distribution, suggesting a complex historical process or long-term stability. In contrast, *H. macroloba* exhibited lower population differentiation, extensive gene flow, and non-significant neutrality test results, indicating long-term demographic stability without recent, drastic population events. Further validation based on gene flow analysis and divergence time estimation revealed that the lineage divergence of *H. discoidea* is older, while *H. macroloba* represents a lineage with a relatively younger evolutionary origin restricted to the Indo-Pacific region. This striking dichotomy clearly illustrates the interplay between intrinsic species-specific traits (e.g., dispersal capacity) and extrinsic historical factors (e.g., paleo-oceanographic events), leading to contrasting evolutionary outcomes among widespread marine taxa. By elucidating how differing evolutionary histories influence patterns of genetic diversity, this study provides a predictive framework for evaluating the resilience and guiding conservation priorities for critical marine calcifiers in the context of rapid environmental change.

## Simple Summary

*Halimeda*, a genus of calcified green macroalgae, is a cornerstone of tropical reef ecosystems; however, the evolutionary mechanisms driving its diversity remain largely underexplored. In this study, we analyzed molecular data from the Xisha (Paracel) Islands to assess *Halimeda* diversity. Focusing on two cosmopolitan species, *Halimeda discoidea* and *Halimeda macroloba*, we uncovered strikingly distinct evolutionary trajectories despite their shared environment. Populations of *H. discoidea* appear genetically fragmented with limited gene flow, indicating an ancient lineage shaped by long-term isolation. In contrast, *H. macroloba* exhibits high connectivity across islands, suggesting a stable demographic history driven by more recent dispersal events. These contrasting patterns demonstrate that both intrinsic species traits and historical oceanographic processes jointly determine population structure. Understanding these unique histories provides critical insights for predicting resilience and prioritizing conservation strategies in the face of rapid climate change.

## 1. Introduction

Marine macroalgae hold significant research importance and application value due to their extensive ecological service functions (e.g., carbon sequestration, biodiversity maintenance) and application potential (including food, pharmaceuticals, and bioenergy sectors) [1,2,3]. The genus *Halimeda* Lamouroux (Bryopsidales, Chlorophyta) is an important calcareous green macroalga that is widely distributed in tropical and subtropical marine regions worldwide [4]. As a significant component of coral reef ecosystems, it is renowned for its massive biomass and rich species diversity, with habitats extending from shallow waters to depths of 90–130 m on coral reef slopes [5]. Additionally, *Halimeda* species can produce secondary metabolites or bioactive molecules with antimicrobial activity, thereby conferring further economic value [6,7]. However, as a calcified alga, *Halimeda* is particularly susceptible to changes in the marine environment, especially ocean acidification [8,9]. Given its significant ecological and economic importance, the genus *Halimeda* has garnered widespread attention regarding its distribution and taxonomy [7,10,11].

Despite the wide geographical distribution and high ecological significance of the *Halimeda* genus, exploration of its diversity, particularly in large-scale coral reef systems such as the Xisha (Paracel) Islands, remains challenged by inherent limitations. On the one hand, certain *Halimeda* species exhibit significant phenotypic variability, i.e., they display variable morphological characteristics under different environmental conditions, which can sometimes lead to morphological misidentification [10,12]. At the same time, the genus is replete with many pseudo-cryptic and cryptic species [13,14]. These features make species definition based solely on morphology both tricky and untrustworthy.

Molecular approaches have thus become an indispensable tool for addressing such taxonomic ambiguities and vagueness [15]. Genetic markers can directly detect genetic differentiation within species, hence more informatively revealing their actual species boundaries and evolutionary relationships. Among various molecular markers, chloroplast DNA markers such as *tuf*A, *rps*3-*rpl*14, and *rbc*L have been widely applied in phylogenetic and phylogeographic studies of macroalgae in the Bryopsidales order owing to their high sequence conservation and stable maternal inheritance [10,11]. The *tuf*A gene sequence, which exhibits moderate variability and high resolution within the genus *Halimeda*, has proven to be the most frequently used molecular marker in deciphering its species evolutionary history and population structure [9,16,17].

Within the context of the aforementioned research background, the current work aims to utilize *tuf*A sequence information to first investigate the species richness of the genus *Halimeda* in the Xisha (Paracel) Islands. Secondly, it will conduct a thorough investigation of the genetic diversity and population structure of two globally widespread *Halimeda* species—*Halimeda discoidea* Decne. and *Halimeda macroloba* Decaisne. By examining their population dynamics and gene exchange patterns across their wide geographical ranges, we aim to provide novel insights into their individual evolutionary histories and biogeography, further elucidating the processes that create diversity within the genus *Halimeda*.

## 2. Materials and Methods

### 2.1. Sample Collection and DNA Extraction

We collected *Halimida* samples in the Xisha (Paracel) Islands in South China Sea in April 2021, with geographical coordinates ranging from 111.2104° to 112.7286° east longitude and 15.79319° to 17.12749° north latitude. The samples are stored at low temperatures and transported to the laboratory for further processing. Total genomic DNA was extracted in the laboratory using the Ezup Spin Column Super Plant Genomic DNA Extraction Kit (Sangon Biotech Co., Ltd., Shanghai, China) in accordance with the manufacturer’s instructions. The quality and concentration of DNA were quantified using a NanoDrop spectrophotometer (Thermo Fisher Scientific, Waltham, MA, USA).

### 2.2. PCR Amplification and Sequencing

The *tuf*A gene sequence was amplified by polymerase chain reaction (PCR) using the primers and amplification protocols [18]. Then the PCR products were sent to Sangon Biotech Co., Ltd. (Shanghai, China) for Sanger sequencing.

### 2.3. Sequence Alignment and Phylogenetic Analysis

The obtained *tuf*A gene sequences were preliminarily aligned by BLAST v2.16.0 (Basic Local Alignment Search Tool) in the National Center for Biotechnology Information (NCBI). The sequences of related species were downloaded, and multi-sequence alignment was performed with the sample sequences of the Xisha (Paracel) Islands using MAFFT v7.526 [19]. Subsequently, manually delete double-ended sequences that cannot be aligned due to different lengths. The phylogenetic tree was constructed in MEGA X v10.2.6 using the maximum likelihood (ML) and the unweighted paired group method with arithmetic mean (UPGMA) algorithm [20].

### 2.4. Haplotype Analysis and Network Construction

Polymorphic locus identification was performed using DnaSP v6.12.03 [21], and haplotype diversity (Hd), nucleotide diversity (π), and the distribution of haplotypes in the population were calculated. A median-joining haplotype network was constructed using PopArt v1.7 [22].

### 2.5. Population Genetic Structure

Molecular analysis of variance (AMOVA), paired fixed index (F_ST_), neutral tests including Tajima’s D and Fu’s FS, and mismatch distribution analysis were all calculated using Arlequin v3.5.2 [23]. Principal coordinate analysis based on genetic distance (PCoA) was performed in R using the “ape” package [24].

### 2.6. Bayesian Clustering

STRUCTURE v2.3.4 is used for Bayesian cluster analysis [25]. Set the number of genetic clusters (K) value from 1 to 10, and repeat each K value 10 times to detect the best K value. The generation number of Markov Chain Monte Carlo (MCMC) is set at 100,000 generations.

### 2.7. Divergence Time Estimation

Using jModelTest v2.1.7 [26], the most suitable nucleotide substitution model GTR+G (General Time Reversible with Gamma distribution) was determined based on the Akaike Information Criterion (AIC). The root age of the tree was set to 147 Ma using BEAST v2.7.7 [27,28]. The divergence time was estimated using a Yule speciation prior and an uncorrelated lognormal relaxed clock model, with the MCMC chain running for 200 million generations [13]. The convergence and effective bit size of MCMC chains were evaluated using Tracer v1.7.2 (ESS > 200) [29]. In TreeAnnotator v2.7.7, the top 10% of trees were discarded as aging trees to generate the maximum evolutionary branch confidence (MCC) tree with an average divergence time and a 95% highest posterior density (HPD) interval. All trees were visualized using FigTree v1.4.4 and iTOL (https://itol.embl.de (accessed on 11 May 2025)).

### 2.8. Gene Flow and Demographic Parameters

Historical gene flow and effective population sizes were estimated using MIGRATE-N v4.4.3 [30]. Heating was set with four temperatures (1.0, 1.5, 2.5, and 5.0) with a static scheme.

## 3. Results

### 3.1. Halimeda Species Diversity in the Xisha (Paracel) Islands: New Discoveries

**Figure 1:**
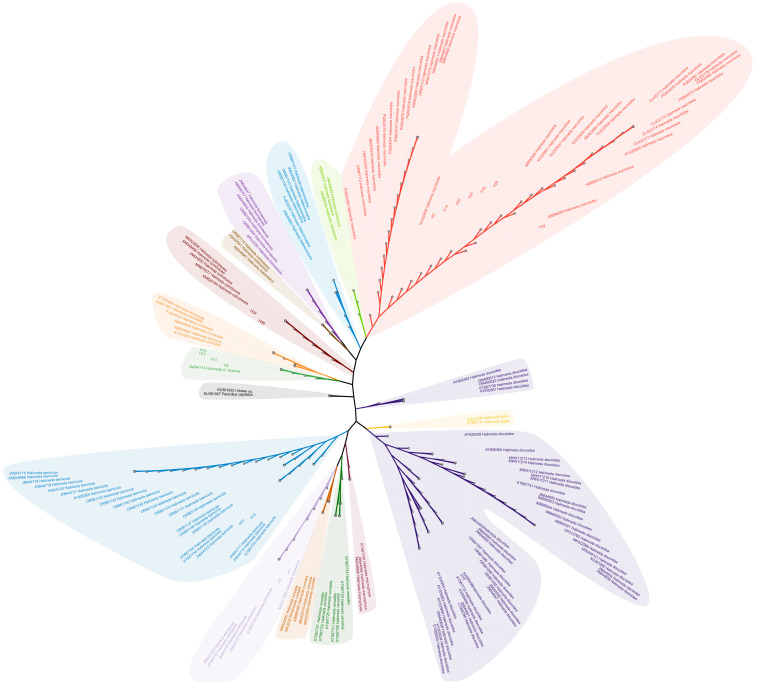
Unrooted maximum likelihood phylogenetic tree constructed based on the *tuf*A gene sequence. The colors of the branches represent different species.

We obtained a total of 17 sequences of *Halimeda* in the Xisha Islands. These sequences were preliminarily compared through BLAST, and sequences of similar species in NCBI with a length greater than 800 bp, as well as the sample sequences, were selected to construct a phylogenetic tree together. The sample sequences consist of 6 species, including *H. macroloba*, *H. discoidea*, *Halimeda cylindracea* Decaisne, *Halimeda xishaensis* C.K.Tseng & M.L.Dong, *Halimeda taenicola* W.R.Taylor, and *Halimeda cf stuposa*. Among them, *H. cylindracea* and *H. cf stuposa* were first discovered in the Xisha (Paracel) Islands by us (Figure 1). In NCBI, *H. macroloba* and *H. discoidea* have the most *tuf*A gene sequences, while other species have fewer sequences. Therefore, due to their higher number of available sequences, we selected these two species for subsequent analysis. In addition, according to the sampling information obtained from NCBI (Table 1), *H. macroloba* is distributed in the Pacific and Indian Oceans, while *H. discoidea* has a wider distribution range and is also found in the Atlantic Ocean. In summary, this preliminary analysis expanded the known *Halimeda* diversity in the region and identified *H. discoidea* and *H. macroloba* as ideal candidates for in-depth population studies (Figure 1; Table 1).

### 3.2. tufA Sequence Characteristics and Haplotype Polymorphism in Halimeda

Due to the differences in primers and sequencing quality, we obtained 41 *H. discoidea* sequences of 608 bp. We also obtained 713 bp sequences from *H. macroloba*. There are significant differences between the two in the number of haplotypes and polymorphic loci (Table 2). *H. discoidea* has 16 haplotypes, with haplotype diversity (Hd) of 0.906 ± 0.027 and nucleotide diversity of 0.02765. However, *H. macroloba* has only four haplotypes, with Hd of 0.523 ± 0.00286 and nucleotide diversity of 0.00089 ± 0.00016. These results indicate that the diversity of *tuf*A sequences in *H. discoidea* is significantly higher than that in *H. macroloba*.

**Figure 2:**
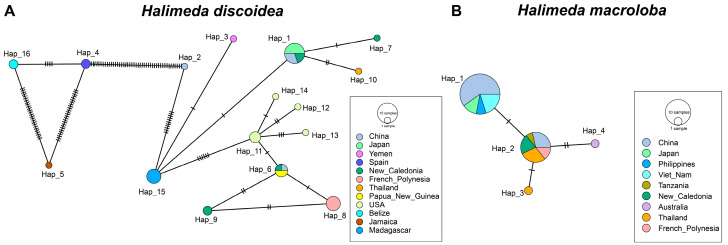
Median-joining haplotype networks of the *tuf*A locus for (**A**) *Halimeda discoidea* and (**B**) *Halimeda macroloba*. Circle fill colors indicate the geographic origin of the haplotypes. The area of each circle is proportional to its haplotype frequency. Perpendicular tick marks on the connecting lines represent the number of mutations between adjacent haplotypes.

Genetic diversity varies geographically, with lower levels observed in some regions (e.g., Japan). *H. discoidea* maintains high diversity in New Caledonia (Hd = 0.867 ± 0.129), the United States (Hd = 0.8000 ± 0.172), and China (Hd = 0.500 ± 0.265). In contrast, *H. macroloba* shows relatively higher diversity in China (Hd = 0.400 ± 0.237) and Thailand (Hd = 0.341 ± 0.111). The haplotype network analysis further reveals that the four haplotypes of *H. macroloba* differ by only one to two base substitutions (Figure 2B). Significant base differences are observed among the *H. discoidea* haplotypes, among which Hap_4, Hap_5, and Hap_16 were particularly divergent from the others (Figure 2A). These three different haplotypes originated, respectively, in Belize, Jamaica, and Spain. Taken together, these findings consistently demonstrate that *H. discoidea* possesses a significantly deeper and more complex genetic diversity compared to *H. macroloba* (Figure 2; Table 2).

### 3.3. Genetic Divergence and Population Structure of H. discoidea

**Figure 3:**
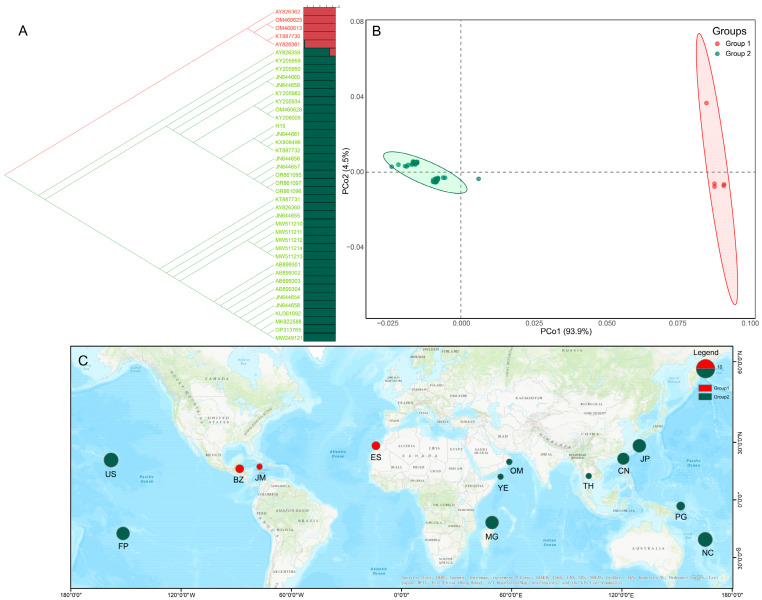
The population genetic structure and global distribution of *Halimeda discoidea*. (**A**) UPGMA tree based on the genetic distance of the *tuf*A gene sequence (left) and Bayesian clustering analysis based on the STRUCTURE software (right). (**B**) Principal Coordinate Analysis (PCoA) reveals genetic differentiation among populations of *H. discoidea*. (**C**) Global geographical distribution of the *H. discoidea* population. In Figures (**A**–**C**), different colors represent cluster groups, with Group 1 being red and Group 2 being green.

To further study the distribution of the *H. discoidea tuf*A gene sequences, we constructed the UPGMA tree based on the genetic distance of all sequences (Figure 3A). *H. discoidea* can be divided into two different groups: Group 1 consists of four sequences from Belize, Jamaica, and Spain, with each country contributing at least one sequence. This grouping is consistent with our previous haplotype analysis. The second group consists of 37 sequences, primarily from China and Australia, but also includes sequences from other regions. The PCoA diagram also shows a clear separation between the two groups, further confirming our findings (Figure 3B).

To visually explore the clustering trends, we additionally performed a Bayesian cluster analysis using STRUCTURE v2.3.4. While recognizing that this software is typically applied to recombining markers, the analysis of our haploid dataset yielded a distinct peak at K = 2. This clustering pattern is highly congruent with the UPGMA tree and PCoA results, serving as a supplementary visualization of the two divergent lineages (Figure 3A,B). All the above results consistently indicate that *H. discoidea* can be divided into two distinct groups. We also visualized the distribution locations of these two groups on the map (Figure 3C). The first group is distributed along the Atlantic coast, while the second group is distributed along the Indian Ocean and the Atlantic coast.

### 3.4. Population Dynamics and Evolutionary History of H. discoidea and H. macroloba

**Figure 4:**
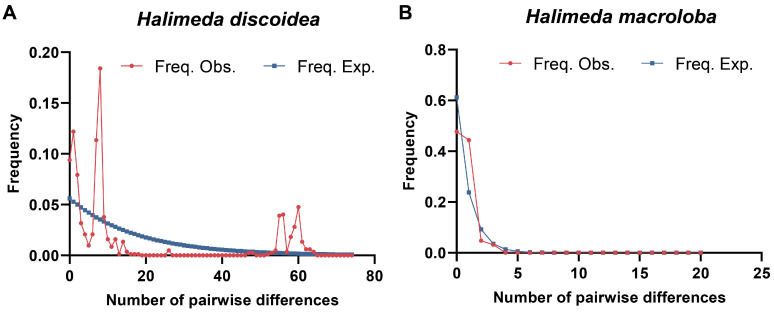
Mismatch distribution analyses for (**A**) *Halimeda discoidea* and (**B**) *Halimeda macroloba* populations. The red line represent the observed frequencies of pairwise nucleotide differences, while the blue line indicates the expected frequencies under a sudden demographic expansion model.

To clarify the population dynamics and evolutionary processes of *H. discoidea* and *H. macroloba*, we utilized the mismatch distribution analysis (Figure 4). The mismatch curve of *H. discoidea* shows a main peak at Diff = 8, supporting a possible population expansion. However, the Ewens-Watterson test did not show significant deviations from the neutral model, indicating no statistical evidence for departure from neutrality (Table 3). Chakraborty’s allele number test also showed no significant departure from the neutral expectation (≈21). Tajima’s D-test yielded a slightly negative but not significant value, which may be due to weak expansion signals or insufficient statistical power caused by a small sample size. Fu’s FS test produced a non-significant positive FS value. The Ewens-Watterson test (*p* = 0.618, *p* > 0.05) indicated that the observed F value (0.11600) was very close to the expected F value (0.11375). These results collectively indicate that there is no strong evidence to support recent population expansion in *H. discoidea*.

For *H. macroloba*, both Tajima’s D (D = −0.76354, *p* = 0.235) and Fu’s FS (FS = −0.42185, *p* = 0.354) yielded negative values. However, none of these values were statistically significant (*p* > 0.05), indicating no significant statistical departure from neutral expectations. This interpretation is further supported by the Ewens-Watterson test (*p* = 0.545), where the observed homozygosity (0.48959) closely aligned with the expected value (0.50546). Similarly, Chakraborty’s test detected no significant deviation from the neutral model (*p* = 0.548). Collectively, these non-significant results do not provide strong statistical evidence for recent bottlenecks or expansions in *H. macroloba*.

### 3.5. Inter-Population Genetic Differentiation (F_ST_) in H. discoidea and H. macroloba

**Figure 5:**
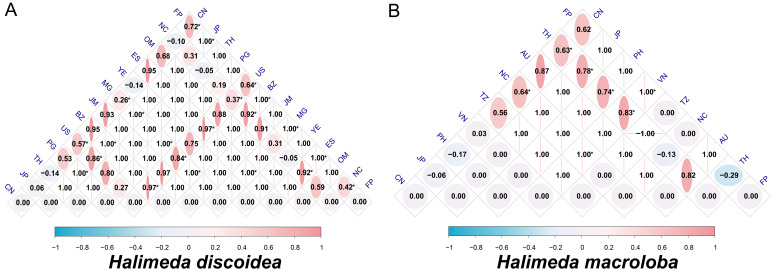
Heatmaps illustrating pairwise F_ST_ values of (**A**) *Halimeda discoidea* and (**B**) *Halimeda macroloba* calculated based on the *tuf*A gene sequences. The color range is from blue (indicating an F_ST_ value close to −1, low genetic differentiation/high genetic similarity) to red (indicating an F_ST_ value close to 1, high differentiation/genetic isolation). An asterisk (*) indicates a statistically significant difference (*p* < 0.05).

To assess inter-population genetic differentiation, we calculated pairwise F_ST_ values for *H. discoidea* and *H. macroloba* based on their sampling locations (Figure 5). *H. discoidea* consists of 13 populations from different locations. The USA (US) population showed significant differentiation from 7 out of the 12 other populations. This was particularly evident with populations from China (CN) (F_ST_ = 0.5665, *p* < 0.05), Belize (BZ) (F_ST_ = 0.97324, *p* < 0.05), and French Polynesia (FP) (F_ST_ = 0.63855, *p* < 0.05). The Belize (BZ), Madagascar (MG), and French Polynesia (FP) populations exhibited significant differentiation from over 50% of the other populations (with at least 6 out of 12 significant pairs). This suggests that these populations serve as significant hubs of genetic differentiation within the species (Figure 5A).

Conversely, populations from Thailand (TH), Jamaica (JM), Yemen (YE), and Oman (OM) did not show significant differentiation with any other population (all F_ST_ values were non-significant). Notably, while the Thailand (TH) population exhibited an F_ST_ of 1.000 (the theoretical maximum) with populations from Japan (JP) and Papua New Guinea (PG), the corresponding *p*-values were not significant (Figure 5A). This suggests that these extreme values might be influenced by small sample sizes or complex evolutionary histories.

Significant differentiation was observed between population pairs such as China (CN)—Madagascar (MG) (F_ST_ = 0.2638) and China (CN)—French Polynesia (FP) (F_ST_ = 0.71798). In contrast, combinations with negative F_ST_ values, such as China (CN)—Thailand (TH) (F_ST_ = −0.14286), were not significant, reflecting complex gene flow patterns among certain populations (Figure 5A).

*H. macroloba* includes nine populations, and its F_ST_ matrix reveals a wide range of variation. Values ranged from a minimum of −0.16684 (China (CN)—Philippines (PH)) to a maximum of 1.000, involving multiple comparisons like Japan (JP)—Tanzania (TZ), Vietnam (VN)—New Caledonia (NC), and Japan (JP)—French Polynesia (FP) (Figure 5B). Negative F_ST_ values (4 out of 9 pairs) may stem from heterozygote excess or small sample size bias.

The Thailand (TH) population exhibited significant differentiation with the China (CN), Japan (JP), Philippines (PH), and Vietnam (VN) populations (4 out of 8 significant pairs; F_ST_ ranging from 0.63196 to 0.83333), indicating a high degree of genetic isolation. The Vietnam (VN) population also showed significant differentiation with multiple populations (New Caledonia (NC), Thailand (TH), French Polynesia (FP); 3 out of 8 significant pairs; F_ST_ from 0.83333 to 1.000). Populations of Tanzania (TZ) and Australia (AU) did not show significant differentiation from any other population. Although the Tanzania (TZ) population had extreme F_ST_ values (e.g., Tanzania (TZ)—Japan (JP) F_ST_ = 1.000), their *p*-values were greater than 0.05 (Figure 5B). These extreme FST values should be interpreted cautiously, as they may result from very small sample sizes rather than reflecting true genetic differentiation.

Overall, *H. discoidea* exhibits more differentiation hubs (6 out of 13 populations vs. 1 out of 9 for *H. macroloba*) and a higher proportion of significantly differentiated pairs (average of 85% for *H. discoidea* hubs vs. 50% for *H. macroloba*) (Figure 5). This implies a more fragmented population structure for *H. discoidea*, possibly due to greater habitat heterogeneity.

### 3.6. Gene Flow and Dispersal Mechanisms in H. discoidea and H. macroloba Across Geographic Regions

**Figure 6:**
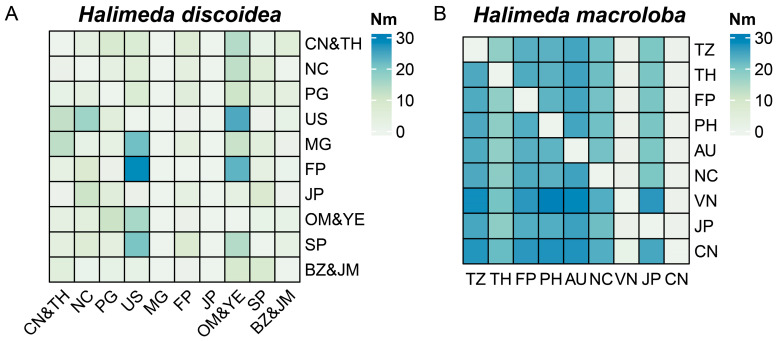
Heatmaps illustrating inter-population gene flow (Nm) derived from *tuf*A sequences for (**A**) *Halimeda discoidea* and (**B**) *Halimeda macroloba*. In each heatmap, gene flow is depicted from the population in the row to the population in the column. The intensity of the color corresponds to the magnitude of gene flow (migration rate), with the scale ranging from 0 to 30. Darker colors indicate higher migration rates.

To better analyze gene flow in *H. discoidea* and *H. macroloba* across different regions, we combined closely related regions with fewer sequence combinations into single populations. Our gene flow analysis revealed significant differences in the dispersal patterns of these two species (Figure 6). Gene exchange in *H. discoidea* has a hierarchical source-sink structure. The Oman-Yemen (OM&YE) region acts as a core for transoceanic gene dispersal, directing the most gene flow towards the USA (US; Nm = 24.35) and French Polynesia (FP; Nm = 23.02). The USA (US) then becomes a central hub, receiving gene flow from the Oman-Yemen (OM&YE; Nm = 24.35) and New Caledonia (NC; Nm = 16.45), and subsequently sending high-intensity gene flow to Madagascar (MG; Nm = 21.20). In contrast, Japan (JP) functions as a naturally isolated node, with its total gene input (ΣNm_in_ = 32.3) being significantly higher than its output (ΣNm_out_ = 1.10) by 29.4 times. The Belize and Jamaica (BZ&JM) region shows marginal connectivity, with average gene flow values below 5, reflecting the impact of natural geographical fragmentation.

For *H. macroloba*, Vietnam (VN) and China (CN) act as terminal gene sinks. Together, they absorb 68% of the total migration, with Vietnam (VN) receiving a total input of ΣNm_in_ = 185.0 and China (CN) receiving ΣNm_in_ = 180.0. This creates a highly one-sided system. A clear example is the absolute unidirectional gene flow from Australia (AU) to China (CN), with a flow rate of 27.54, while the reverse flow is 0. Similarly, the gene flow from the Philippines (PH) to Vietnam (VN) (Nm = 29.79) is 27.6 times greater than the reverse flow. In contrast, the gene flow from China (CN) to Vietnam (VN) (Nm = 1.29) has no reverse flow from Vietnam to China (VN → CN = 0). These distinct gene flow patterns highlight the differing strategies and geographic influences on the dispersal of *H. discoidea* and *H. macroloba*.

### 3.7. Phylogenetic Relationships and Divergence Times of Halimeda Species

**Figure 7:**
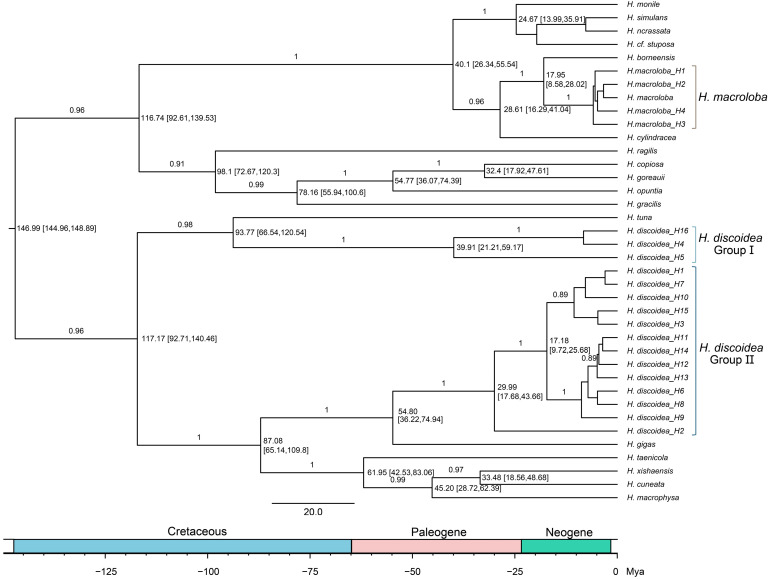
Bayesian phylogenetic tree constructed using *tuf*A gene haplotype sequences. Node values indicate Bayesian posterior probabilities (BPP), while branch lengths are scaled to estimated divergence times (in millions of years ago, Ma), with 95% highest posterior density (HPD) intervals provided at each node.

The Bayesian phylogenetic tree (Figure 7), constructed using *tuf*A gene sequences, illustrates the evolutionary relationships and divergence times among *Halimeda* species. The Bayesian phylogenetic tree strongly supports the monophyly of *H. macroloba* and *H. discoidea* clades. Within *H. discoidea*, two well-supported, distinct lineages were identified: Group I and Group II (BPP = 0.96), confirming the significant phylogenetic differentiation within this species as observed in previous analyses. The divergence between *H. discoidea* Group I and Group II is estimated to have occurred in the Cretaceous period, around 117.17 Ma (95% HPD: 92.71–140.46). *H. discoidea* Group I, encompassing haplotypes H4, H5, H16, H7, H10, H15, H3, H11, and H13, shows a deeper divergence from *H. tuna* at 93.77 Ma (95% HPD: 66.54–120.54). Furthermore, *H. discoidea* Group II, which includes haplotypes H1, H2, H6, H8, H9, and H12, diverged from *H. gigas* more recently, approximately 54.80 Ma (95% HPD: 36.22–74.94). Internal nodes within *H. discoidea* also show recent diversification events. H16, H7, H10, H15, H3, H11, and H13 at 39.91 Ma (95% HPD: 21.21–59.17), and the divergence within Group II at 17.18 Ma (95% HPD: 3.72–25.68) and 29.99 Ma (95% HPD: 17.63–43.66) (Figure 7).

For *H. macroloba*, the analysis indicates a relatively recent evolutionary history within the clade. The *H. macroloba* clade, comprising haplotypes H1-H4, diverged from *H. borneensis* at 17.95 Ma (95% HPD: 8.58–28.02). Other closely related species, such as *H. cylindracea*, *H. ragilis*, *H. copiosa*, *H. goreaui*, and *H. opuntia*, are positioned as sister taxa to the *H. macroloba*/*H. borneensis* clade, with their common ancestor dating back to 40.1 Ma (95% HPD: 26.34–55.54) (Figure 7).

The Bayesian phylogenetic tree also depicts the phylogenetic positions of other *Halimeda* species, including *H. monile*, *H. simulans*, *H. gracilis*, *H. incrassata*, *H.* cf. *stuposa*, *H. xishaensis*, *H. taenicola*, *H. cuneata*, and *H. macrophysa*, thereby offering a comprehensive view of the evolutionary relationships within the genus *Halimeda*. The basal divergence of *H. xishaensis* and *H. taenicola* from other clades is estimated to have occurred approximately 61.95 million years ago (95% HPD: 42.53–83.06), as shown in Figure 7. Consequently, this Bayesian phylogenetic tree not only illustrates the ancient and complex global lineage history of *H. discoidea* compared to the regionally restricted *H. macroloba,* but also establishes a broader evolutionary framework for the entire genus.

## 4. Discussion

### 4.1. Halimeda Biogeography in the Xisha (Paracel) Islands: Insights from New Molecular Records

Due to challenges in sample collection, the diversity of calcified algae in the Xisha (Paracel) Islands remains insufficiently studied [31]. Phylogenetic reconstruction based on *tuf*A gene sequences (Figure 1) not only clarified phylogenetic relationships among previously known *Halimeda* species but also identified new records of *H. taenicola* and *H.* cf. *stuposa* in the waters of the Xisha (Paracel) Islands. *H.* cf. *stuposa* was initially discovered in New Caledonia through *tuf*A gene sequences and was assigned an identifier based on morphological congruence with *H. stuposa* [11]; since its initial discovery, the species has been rarely documented. This finding expands the known local species diversity of the genus and indicates that biodiversity assessments relying solely on traditional morphological methods may underestimate algal biodiversity in the Xisha (Paracel) Islands and other underexplored marine regions.

The geographical distribution patterns of species within the genus *Halimeda* exhibit considerable variation [32]. Among them, *H. discoidea*, *H. macroloba*, *H. opuntia*, and *H. tuna* are considered common species due to their relatively widespread presence in the South China Sea [12,16,33,34]. Notably, *H. discoidea* has an exceptionally broad distribution that extends into the Atlantic Ocean and adjacent islands, whereas *H. macroloba* is predominantly found in the vicinity of islands in the Pacific and Indian Oceans. In contrast, *H. taenicola* is a rare species. It was previously documented in Vietnamese waters [35,36] and more recently in Malaysia [37]. The Indo-Pacific region, which includes the Xisha (Paracel) Islands, is known for its high species richness of *Halimeda*, with as many as 25 species recorded [35]. However, only 13 species have been reported in Chinese waters to date [38], suggesting that the *Halimeda* diversity in the South China Sea region of China remains underexplored and merits further investigation.

### 4.2. Contrasting Population Structures: High Fragmentation in H. Discoidea Vs. Relative Connectivity in H. macroloba

Our results illustrate striking differences in population genetic structure between *H. discoidea* and *H. macroloba*, even where their geographic distributions are prone to overlap. *H. discoidea* exhibited an extremely disjunctive population structure, with more centers of differentiation (6 out of 13 populations) and a higher proportion of highly differentiated pairwise comparisons (averaging 85%). This pattern was exemplified by populations from the USA, Belize, Madagascar, and French Polynesia, which showed extremely high differentiation from over 50% of other sampled populations. Specific examples, such as the extreme differentiation observed between China and Madagascar (F_ST_ = 0.2638) and China and French Polynesia (F_ST_ = 0.71798), further reflect this pattern of high genetic isolation. Additionally, *H. discoidea* is typically divided into two groups, which may be due to the north–south orientation of the African and American continents, preventing tropical marine organisms from spreading between the Atlantic and Indo-Pacific basins [14].

In contrast, *H. macroloba* displayed a relatively less fragmented structure, consistent with previous studies based on multiple molecular markers [34,36,39]. In this case, only the Thai population acted as a major center of genetic isolation, strongly differentiated from four out of eight other populations (Figure 5B).

These contrasting patterns are most likely the result of species-specific dispersal capacities, life cycles, and responses to environmental heterogeneity [40,41]. For *H. discoidea*, high differentiation with multiple hubs evidently reveals limited gene flow and strong geographical isolating forces [14,42]. The persistently non-significant F_ST_ values observed in populations such as those from Thailand, Jamaica, Yemen, and Oman require further scrutiny, as they may indicate unique regional dynamics or simply reflect limitations in current sampling. The occurrence of negative F_ST_ values (e.g., for the China–French Polynesia pair in *H. discoidea*), although not significant, implies complex gene flow patterns that may be influenced by small sample size biases and complicate straightforward interpretations of population connectivity.

### 4.3. Divergent Gene Flow Regimes Explain Contrasting Genetic Structures

The gene flow plot (Figure 6) illustrates both the direction (Nm values) and magnitude of migration among different populations, thereby revealing the underlying mechanisms driving variation in population genetic structure. The gene flow heatmap indicates that *H. macroloba* exhibits more extensive and higher-intensity gene flow (Figure 6B), with Nm values among most population pairs significantly larger than those observed in *H. discoidea*. For instance, high Nm values were observed for gene flow from Thailand (TH) to Australia (AU) and from the Philippines (PH) to Australia (AU), reflecting intensive gene flow in these directions. This pronounced gene flow likely accounts for the relatively lower F_ST_ values and less fragmented genetic structure of *H. macroloba*, which is consistent with signs of demographic expansion [43]. In contrast, gene flow intensity in *H. discoidea* appears to be generally weaker and more restricted (Figure 6A). Although some moderate gene flow pathways exist (e.g., from China/Thailand to the USA, or from Madagascar to French Polynesia), most Nm values for most population pairs are close to zero. This restricted gene flow directly contributes to the strongly differentiated population structure of *H. discoidea* [44,45].

### 4.4. Deep Evolutionary Divergence Explains Contrasting Modern Population Structures

Based on the tree topology and divergence time estimation (Figure 7), the divergence time between Group I and Group II within *H. discoidea* was estimated at 117.17 Ma (95% HPD: 92.71–140.46 Ma), during the Cretaceous period. This suggests that the lineages of *H. discoidea* diversified during a relatively ancient period, possibly driven by geological and marine environmental changes at that time. However, this divergence timing does not align with the geological events typically used to explain the sister relationship between the strictly Atlantic and strictly Indo-Pacific lineages. These events include: (1) the expansion of the Atlantic Ocean, which began during the Jurassic period (±170–160 Ma) [46]; (2) the collision of the Afro-Arabian Plate and the Eurasian Plate during the Miocene (±15–12 Ma) [47] (Rögl & Steininger, 1984); and (3) the closure of the Central American Seaway during the Pliocene (±3 Ma) [48]. These events do not match the divergence timeline of *H. discoidea*.

An important oceanographic event during the Cretaceous period may have limited gene flow between the Atlantic and Indo-Pacific basins and caused the equatorial ocean current, which homogenizes tropical marine communities, to shift toward southern Africa [49]. This result suggests that geological barriers may not have been the initial cause of speciation but rather acted as reinforcing barriers after oceanographic events triggered speciation [42]. Similar conclusions have been drawn from molecular and paleontological studies of species in the Central American Isthmus [50]. The universality of this pattern requires further investigation.

For *H. macroloba*, its lineage’s MRCA split approximately 28.61 Ma ago (95% HPD: 16.29–41.04 Ma). This split is younger than the internal lineage split of *H. discoidea*, which occurred during the late Paleogene to early Neogene. This result aligns with the status of *H. macroloba* as an Indo-Pacific endemic species. The younger divergence time suggests that it has undergone a relatively recent diversification within this basin, rather than developing ancient and strongly differentiated global lineages like *H. discoidea*.

### 4.5. Limitations and Future Perspectives

While this study presents valuable information, several limitations need to be considered. One key limitation is the sole reliance on a single genetic marker (*tuf*A), which may not fully capture the evolutionary history or fine-scale population structure properly. This is particularly problematic in cases of incomplete lineage sorting or hybridization. Additionally, high F_ST_ values observed in some populations with non-significant p-values (e.g., high F_ST_ values ranging from 0.8742 to 1.000 between *H. macroloba* in Jamaica and other regions, and between *H. discoidea* in Australia and other regions) could be influenced by small sample sizes, highlighting limitations in statistical power for these comparisons. The presence of *H. discoidea* in the Atlantic, in contrast to the strictly Indo-Pacific distribution of *H. macroloba*, may lead to the observation of higher genetic diversity and older divergence time estimates in the former. Consequently, direct comparisons of diversity and evolutionary age should be interpreted with caution, considering these distinct global versus regional biogeographic contexts.

Future studies should incorporate more unlinked genetic markers, such as nuclear microsatellites or genomic-scale markers (e.g., Restriction-site Associated DNA sequencing (RAD-seq), whole-genome sequencing), to achieve higher resolution in population connectivity and demographic inference [51,52]. Expanding the geographical sampling to fill the gaps will further elucidate species distribution and gene flow dynamics. Moreover, integrating molecular data with ecologically relevant information, such as dispersal capabilities, reproductive modes, and habitat occupancy, will be crucial for a deeper understanding of the mechanisms underlying the differing population structures in *H. discoidea* and *H. macroloba* [53,54,55]. Such integrated approaches will facilitate more effective conservation actions for these important marine calcifiers in a changing ocean.

## 5. Conclusions

This study reveals novel *Halimeda* diversity in the Xisha (Paracel) Islands, identifying two new records, *H. cylindracea* and *H.* cf. *stuposa*, through chloroplast *tuf*A sequencing. These findings significantly expand the regional species inventory and highlight the power of molecular diagnostics in uncovering cryptic biodiversity in under-explored marine regions. Furthermore, comparative population genetics uncovered divergent evolutionary trajectories for two widespread species. *H. discoidea* displays a highly fragmented population structure and deep global lineage divergence, indicative of a history shaped by profound inter-oceanic geographic isolation. In contrast, *H. macroloba* exhibits lower differentiation and widespread gene flow, consistent with its more recent regional diversification restricted to the Indo-Pacific basin. Together, these results demonstrate that species-specific traits and historical contingencies jointly drive the evolution of cosmopolitan marine taxa. This work establishes a conceptual framework for *Halimeda* diversification and provides a robust foundation for future genomic studies on adaptive evolution and conservation prioritization.

## Figures and Tables

**Table 1:** *Halimeda discoidea* and *Halimeda macroloba* samples information used for phylogenetic and population genetic analysis.

Symbol	Area	Species	GenBank Information	Source	Collection Locality
CN	China	*Halimeda discoidea*	H15	This study	Xisha (Paracel) Islands, Sansha, Hainan
		*Halimeda discoidea*	OP313765	NCBI	Taiwan: Kenting National Park
		*Halimeda discoidea*	MK922588	NCBI	Taiwan: Kenting National Park
		*Halimeda discoidea*	MW249121	NCBI	Taiwan
		*Halimeda macroloba*	H2	This study	Xisha (Paracel) Islands, Sansha, Hainan
		*Halimeda macroloba*	H22	This study	Xisha (Paracel) Islands, Sansha, Hainan
		*Halimeda macroloba*	H24	This study	Xisha (Paracel) Islands, Sansha, Hainan
		*Halimeda macroloba*	H25	This study	Xisha (Paracel) Islands, Sansha, Hainan
		*Halimeda macroloba*	H28	This study	Xisha (Paracel) Islands, Sansha, Hainan
		*Halimeda macroloba*	H32	This study	Xisha (Paracel) Islands, Sansha, Hainan
		*Halimeda macroloba*	PQ824575	NCBI	Pratas Island
		*Halimeda macroloba*	KU220841	NCBI	Pratas Island
		*Halimeda macroloba*	KU220837	NCBI	Pratas Island
		*Halimeda macroloba*	KU220838	NCBI	Pratas Island
		*Halimeda macroloba*	KU220839	NCBI	Pratas Island
		*Halimeda macroloba*	KU220840	NCBI	Pratas Island
		*Halimeda macroloba*	KU220836	NCBI	Pratas Island
		*Halimeda macroloba*	MK922585	NCBI	China
		*Halimeda macroloba*	PQ824576	NCBI	China
		*Halimeda macroloba*	MK922586	NCBI	Taiwan: Taiping Island
		*Halimeda macroloba*	MN879375	NCBI	Taiwan: Taiping Island
		*Halimeda macroloba*	MN879366	NCBI	Taiwan: Taiping Island
		*Halimeda macroloba*	MN879378	NCBI	Taiwan: Taiping Island
JP	Japan	*Halimeda discoidea*	AB899303	NCBI	Amami I., Kagoshima, Japan
		*Halimeda discoidea*	AB899304	NCBI	Ankyaba, Amami I., Kagoshima, Japan
		*Halimeda discoidea*	AB899302	NCBI	Amami I., Kagoshima, Japan
		*Halimeda discoidea*	AB899301	NCBI	Sani, Amami I., Kagoshima, Japan
		*Halimeda discoidea*	KU361892	NCBI	Kurima Island, Miyako
		*Halimeda macroloba*	AB899309	NCBI	Uken, Amami I., Kagoshima, Japan
		*Halimeda macroloba*	AB899308	NCBI	Shirahama, Amami I., Kagoshima
		*Halimeda macroloba*	AB899310	NCBI	Shiraho, Ishigaki I., Okinawa
TH	Thailand	*Halimeda discoidea*	KT887731	NCBI	Thailand
		*Halimeda macroloba*	PQ824574	NCBI	Thailand
		*Halimeda macroloba*	PQ824577	NCBI	Thailand
		*Halimeda macroloba*	PQ824579	NCBI	Thailand
		*Halimeda macroloba*	PQ824578	NCBI	Thailand
		*Halimeda macroloba*	PQ824580	NCBI	Thailand
FP	French Polynesia	*Halimeda discoidea*	OR861097	NCBI	French Polynesia: Society Islands, Moorea
		*Halimeda discoidea*	OR861096	NCBI	French Polynesia: Society Islands, Moorea
		*Halimeda discoidea*	OR861095	NCBI	French Polynesia: Society Islands, Moorea
		*Halimeda discoidea*	JN644657	NCBI	French Polynesia: Moorea
		*Halimeda discoidea*	JN644656	NCBI	French Polynesia: Moorea
		*Halimeda macroloba*	OR861113	NCBI	French Polynesia: Society Islands, Tahiti
		*Halimeda macroloba*	OR861112	NCBI	French Polynesia: Society Islands, Tahiti
PG	Papua New Guinea	*Halimeda discoidea*	KX808496	NCBI	Papua New Guinea
		*Halimeda discoidea*	KT887732	NCBI	Papua New Guinea
MG	Madagascar	*Halimeda discoidea*	MW511210	NCBI	Madagascar: Antsiranana Bay, Baie de Tonnerre
		*Halimeda discoidea*	MW511214	NCBI	Madagascar: Antsiranana Bay, Orangea
		*Halimeda discoidea*	MW511213	NCBI	Madagascar: Antsiranana Bay, Petite passe Orangea
		*Halimeda discoidea*	MW511212	NCBI	Madagascar: Antsiranana Bay, Orangea
		*Halimeda discoidea*	MW511211	NCBI	Madagascar: Antsiranana Bay, Orangea
US	USA	*Halimeda discoidea*	KY206005	NCBI	Oahu, Hunakai Beach
		*Halimeda discoidea*	KY205959	NCBI	Oahu, Hunakai Beach
		*Halimeda discoidea*	KY205982	NCBI	Oahu, Hunakai Beach
		*Halimeda discoidea*	KY205950	NCBI	Oahu, Hunakai Beach
		*Halimeda discoidea*	KY205934	NCBI	Oahu, Hunakai Beach
		*Halimeda discoidea*	OM460628	NCBI	Kaneohe Bay, Kapaka Island, intertidal zone
NC	New Caledonia	*Halimeda discoidea*	JN644661	NCBI	New Caledonia
		*Halimeda discoidea*	JN644660	NCBI	New Caledonia
		*Halimeda discoidea*	JN644659	NCBI	New Caledonia
		*Halimeda discoidea*	JN644658	NCBI	New Caledonia
		*Halimeda discoidea*	JN644655	NCBI	New Caledonia: Chesterfield
		*Halimeda discoidea*	JN644654	NCBI	New Caledonia
		*Halimeda macroloba*	JN644691	NCBI	New Caledonia
		*Halimeda macroloba*	JN644692	NCBI	New Caledonia
		*Halimeda macroloba*	JN644693	NCBI	New Caledonia
OM	Oman	*Halimeda discoidea*	AY826359	NCBI	Oman
JM	Jamaica	*Halimeda discoidea*	AY826362	NCBI	Jamaica
YE	Yemen	*Halimeda discoidea*	AY826360	NCBI	Yemen: Socotra
SP	Spain	*Halimeda discoidea*	AY826361	NCBI	Gran Canaria
		*Halimeda discoidea*	KT887730	NCBI	Fuerteventura
BZ	Belize	*Halimeda discoidea*	OM460625	NCBI	Carrie Bow Cay
		*Halimeda discoidea*	OM460613	NCBI	Carrie Bow Cay
VN	Viet Nam	*Halimeda macroloba*	OL422177	NCBI	Con Dao
		*Halimeda macroloba*	OL422174	NCBI	Viet NaM
		*Halimeda macroloba*	OL422175	NCBI	Ninh Thuan
		*Halimeda macroloba*	OL422173	NCBI	Nha Trang
		*Halimeda macroloba*	OL422176	NCBI	Phy Quy
PH	Philippines	*Halimeda macroloba*	PQ824581	NCBI	Philippines
		*Halimeda macroloba*	PQ824582	NCBI	Philippines
AU	Australia	*Halimeda macroloba*	HM140244	NCBI	Lizard Island, Coconut Beach
TZ	Tanzania	*Halimeda macroloba*	AM049960	NCBI	Zanzibar, Nungwi

**Table 2:** *tuf*A-based haplotype distribution and genetic diversity in *Halimeda discoidea* and *Halimeda macroloba* populations.

Symbol	Country/Region	Species									
		*Halimeda discoidea*					*Halimeda macroloba*				
		Number of Samples (n)	Polymorphism Sites (S)	Haplotype (Sample Numbers)	Haplotype Diversity (Hd)	Nucleotide Diversity (π/Pi)	Number of Samples (n)	Polymorphism Sites (S)	Haplotype (Sample Numbers)	Haplotype Diversity (Hd)	Nucleotide Diversity (π/Pi)
CN	China	4	8	H1(3), H6(1)	0.500 ± 0.265	0.00658 ± 0.265	19	1	H1(15), H2(4)	0.341 ± 0.111	0.00049 ± 0.00016
JP	Japan	5	0	H1(5)	0	0	3	0	H1(3)	0	0
TH	Thailand	1	0	H10(1)	0	0	5	1	H2(4), H3(1)	0.400 ± 0.237	0.00056 ± 0.00033
FP	French Polynesia	5	0	H8(5)	0	0	2	0	H2(2)	0	0
PG	Papua New Guinea	2	0	H6(2)	0	0	-	-	-	-	-
MG	Madagascar	5	0	H15(5)	0	0	-	-	-	-	-
US	USA	6	6	H11(3), H12(1), H13(1), H14(1)	0.8000 ± 0.172	0.00329 ± 0.00113	-	-	-	-	-
NC	New Caledonia	6	10	H1(2), H6(1), H7(1), H9(2)	0.867 ± 0.129	0.00888 ± 0.00168	3	0	H2(3)	0	0
OM	Oman	1	0	H2(1)	0	0	-	-	-	-	-
JM	Jamaica	1	0	H5(1)	0	0	-	-	-	-	-
YE	Yemen	1	0	H3(1)	0	0	-	-	-	-	-
ES	Spain	1	0	H4(2)	0	0	-	-	-	-	-
BZ	Belize	2	0	H16(2)	0	0	-	-	-	-	-
VN	Viet NaM	-	-	-	-	-	5	0	H1(5)	0	0
PH	Philippines	-	-	-	-	-	2	0	H1(2)	0	0
AU	Australia	-	-	-	-	-	1	0	H4(1)	0	0
TZ	Tanzania	-	-	-	-	-	1	0	H2(1)	0	0
Total	Total	41	81	-	0.906 ± 0.027	0.02765 ± 0.00686	41	4	-	0.523 ± 0.00286	0.00089 ± 0.00016

**Table 3:** Results of genetic and mismatch analyze in *Halimeda discoidea* and *Halimeda macroloba*.

Species	Number of Samples	Fu’s Fs, *p*-Value	Tajima’s D, *p*-Value	Ewens-Watterson Test (Obs/Exp), *p*-Value	Chakraborty’s Test
*Halimeda discoidea*	41	3.56909, 0.91300	−0.40625, 0.32600	0.11600/0.11375, 0.618	0.45563
*Halimeda macroloba*	41	−0.42185, 0.35400	−0.76354, 0.23500	0.48959/0.050546, 0.545	0.54814

## Data Availability

Data will be made available on request.
